# In this Issue

**DOI:** 10.1111/cas.14966

**Published:** 2022-12-13

## BRCA1 transports the DNA damage signal for CDDP‐induced centrosome amplification through the centrosomal Aurora A


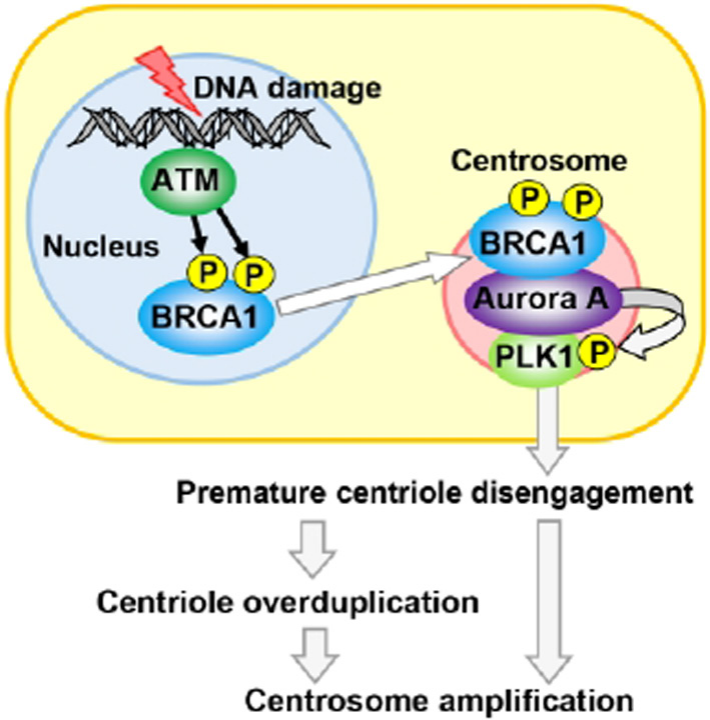


Extensive DNA damage can cause changes to centrosomes—structures in the cytoplasm that prevent cell replication and the continuation of defective cell lines. The process, called DNA damage‐induced centrosome amplification (DDICA), is an emergency mechanism that prevents tumor formation.

Breast cancer gene 1 (BRCA1) is a well‐known tumor suppressor. Mutations in the gene have been linked to the increased risk of breast cancer and ovarian cancer. Although BRCA1 is closely involved in the DDICA process, the exact mechanism by which it causes changes to centrosomes remains unknown.

Qi et al. examined BRCA1 accumulation on centrosomes during the cell cycle after they used the DNA crosslinker cisplatin (CDDP) to induce DNA damage.

Their study found that the centrosomal localization of BRCA1 increased markedly following damage to the DNA. This had two noteworthy consequences—an increase in the localization of the enzyme Aurora A and the activation of the enzyme PLK1 by Aurora A in the centrosome. It is known that the overactivation of PLK1 in turn affects centrosome maturation.

They also found that concentrations of BRCA1 and Aurora A in the centrosome following DNA damage were significantly lowered when they performed the same experiment with cells expressing BRCA1 variants from a patient with cancer. This suggests that BRCA1 variants in patients with cancer fail to produce the DNA damage signal that triggers the BRCA1‐Aurora A‐PLK1 pathway.

Damage to DNA is detected by ataxia telangiectasia mutated (ATM), a sensory protein that modifies BRCA1. As a result of this modification, BRCA1 seems to traverse the nuclear membrane and diffuse into the cytoplasm before entering the centrosome.

The pathway uncovered by their study suggests that BRCA1 acts as an intracellular messenger. By travelling from the nucleus to the cytoplasm and affecting changes to the centrosome through Aurora A and PLK1, BRCA1 can arrest cell replication. Mutant BRCA1 in patients with cancer, however, seems to be incapable of such signaling. This study shows that, during DDICA, BRCA1 has a crucial role in centrosome regulation and DNA repair.

https://onlinelibrary.wiley.com/doi/full/10.1111/CAS.15573

## Cancerous pH‐responsive Polycarboxybetaine‐coated Lipid Nanoparticle for Smart Delivery of siRNA against Subcutaneous Tumor Model


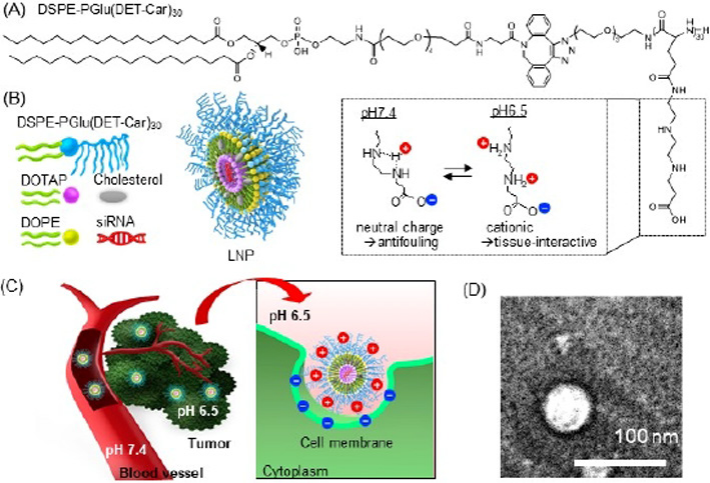


Small interfering RNAs (siRNAs) are used for the treatment of multiple diseases, including cancer. Notably, siRNAs are susceptible to degradation inside cells due to their physiochemical properties, making their delivery difficult. Hence, lipid nanoparticles (LNPs) are generally used as carriers for the systemic delivery of siRNAs, due to their biocompatibility, low‐toxicity, and ability to encapsulate siRNA.

The external surfaces of LNPs play a key role in differentiating between a normal physiological environment and a cancerous environment. An ideal LNP system should be able to recognize tumor‐specific conditions and deliver the required siRNA to the targeted tumor. Such LNPs typically consist of a pH‐responsive lipid which identifies the tumor environment, and a polyethylene glycol (PEG) lipid, which encapsulates the siRNA and delivers it to the target site. At present, these LNPs lack the ability to deliver siRNAs to tissues and organs other than the liver.

In this issue, Sung et al. designed an LNP through surface modifications which allow the molecule to remain neutrally charged at a physiological pH of 7.4, but switch to a positive or cationic charge upon sensing the acidic pH of cancerous tissues in its vicinity. They hoped that the positive charge of the LNPs would allow them to target anionic cancerous tissues with high efficacy.

To achieve this, they used an “ethylenediamine‐based polycarboxybetain” molecule as a “smart shell” for the LNPs, which demonstrated the ability to switch to cationic charge in response to acidic pH. This polycarboxybetain‐modified LNP could seamlessly circulate through the blood at physiological pH, while enhancing cellular uptake of siRNA in cancerous tissues and delivering required nucleic acids to solid tumors.

In addition, it effectively targeted tumor cells in two in‐vitro tumor models, leading to tumor growth inhibition.

Overall, this study demonstrates the potential of pH‐responsive polycarboxybetaine as a surface coating molecule for LNP, which can facilitate the effective and targeted delivery of nanomedicines like siRNAs. The polycarboxybetain‐modifed LNP designed by the authors offers promise as a delivery vehicle in the treatment of cancers and other inflammatory diseases that are characterized by an acidic microenvironment.

https://onlinelibrary.wiley.com/doi/full/10.1111/CAS.15554

## TIMP1 Promotes Cell Proliferation and Invasion Capability of Right‐sided Colon Cancers via the FAK/Akt Signaling Pathway


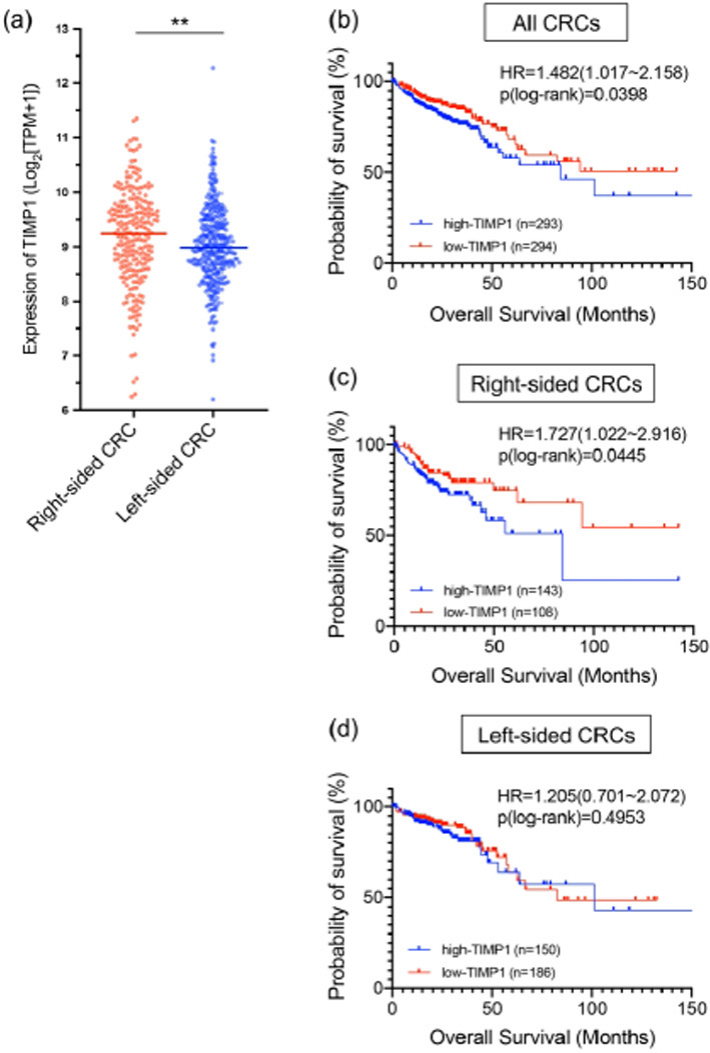


Colorectal cancer (CRC) affects the large intestine and the rectum and is a major cause of cancer‐related deaths. Patients with late‐stage CRC have a poor prognosis and dismal survival outcomes. Notably, it has been observed that the survival rates of patients with right‐sided CRCs are lower as compared to those with left‐sided CRCs. Although scientists have uncovered several differences between right‐sided and left‐sided CRCs, the mechanisms underlying the poorer outcomes in patients with right‐sided CRCs are not well understood.

Recently, Ma et al. compared the cell multiplication and invasion capabilities of left‐sided and right‐sided CRCs by observing patient‐derived organoids (PDO), which are in vitro three‐dimensional cultures of intestinal cells. Additionally, they compared the expression of 184 genes that have been linked to CRC prognosis.

Their experiments revealed that cancer cells derived from right‐sided CRC PDOs demonstrated significantly higher proliferation and invasion than left‐sided CRC PDOs. Gene expression analysis further established that the expression of the tissue inhibitor matrix metalloproteinase 1 (TIMP1) gene was the highest in right‐sided CRC PDOs. Interestingly, lowering the expression of TIMP1 led to a decrease in the cell proliferation and invasion capabilities of right‐sided CRC PDOs. The researchers also observed that TIMP1 exerts its effects by regulating the expression of two proteins—pFAK and pAkt—via the FAK/Akt/Bad signaling pathway.

Further corroborating the experimental findings of this study, the researchers analysed The Cancer Genome Atlas database and found that patients with higher levels of *TIMP1* had a significantly shorter survival than those with lower levels of *TIMP1*. These findings are also consistent with previous reports on the role of TIMP1 in other types of cancers.

Overall, this study offers novel molecular insights into the differences between right‐sided and left‐sided CRC, and sheds light on possible causative factors underlying the poor outcomes in patients with right‐sided CRC. Furthermore, it highlights potential therapeutic targets that may help improve the survival rates of patients with late‐stage right‐sided CRC.

https://onlinelibrary.wiley.com/doi/full/10.1111/CAS.15567

